# The Role of LDH Serum Levels in Predicting Global Outcome in HCC Patients Undergoing TACE: Implications for Clinical Management

**DOI:** 10.1371/journal.pone.0032653

**Published:** 2012-03-26

**Authors:** Mario Scartozzi, Luca Faloppi, Maristella Bianconi, Riccardo Giampieri, Elena Maccaroni, Alessandro Bittoni, Michela Del Prete, Cristian Loretelli, Laura Belvederesi, Gianluca Svegliati Baroni, Stefano Cascinu

**Affiliations:** 1 Clinica di Oncologia Medica, Azienda Ospedaliero-Universitaria “Ospedali Riuniti Umberto I - Lancisi - Salesi”, Università Politecnica delle Marche, Ancona, Italy; 2 Scuola di Specializzazione in Oncologia Medica, Clinica di Oncologia Medica, Università Politecnica delle Marche, Ancona, Italy; 3 Clinica di Gastroenterologia, Azienda Ospedaliero-Universitaria “Ospedali Riuniti Umberto I - Lancisi - Salesi”, Università Politecnica delle Marche, Ancona, Italy; Karolinska Institutet, Sweden

## Abstract

In many tumor types serum lactate dehydrogenase (LDH) levels is an indirect marker of tumor hypoxia, neo-angiogenesis and worse prognosis. However data about hepatocellular carcinoma (HCC) are lacking in the clinical setting of patients undergoing transarterial-chemoembolization (TACE) in whom hypoxia and neo-angiogenesis may represent a molecular key to treatment failure. Aim of our analysis was to evaluate the role of LDH pre-treatment levels in determining clinical outcome for patients with HCC receiving TACE. One hundred and fourteen patients were available for our analysis. For all patients LDH values were collected within one month before the procedure. We divided our patients into two groups, according to LDH serum concentration registered before TACE (first: LDH≤450 U/l 84 patients; second: LDH>450 U/l 30 patients). Patients were classified according to the variation in LDH serum levels pre- and post-treatment (increased: 62 patients vs. decreased 52 patients). No statistically significant differences were found between the groups for all clinical characteristics analyzed (gender, median age, performance status ECOG, staging systems). In patients with LDH values below 450 U/l median time to progression (TTP) was 16.3 months, whereas it was of 10.1 months in patients above the cut-off (p = 0.0085). Accordingly median overall survival (OS) was 22.4 months and 11.7 months (p = 0.0049). In patients with decreased LDH values after treatment median TTP was 12.4 months, and median OS was 22.1 months, whereas TTP was 9.1 months and OS was 9.5 in patients with increased LDH levels (TTP: p = 0.0087; OS: p<0.0001). In our experience, LDH seemed able to predict clinical outcome for HCC patients undergoing TACE. Given the correlation between LDH levels and tumor angiogenesis we can speculate that patients with high LDH pretreatment levels may be optimal candidates for clinical trial exploring a multimodality treatment approach with TACE and anti-VEGF inhibitors in order to improve TTP and OS.

## Introduction

Hepatocellular carcinoma (HCC) represents the commonest primary cancer of the liver. Incidence is increasing and HCC has risen to become the 5th commonest malignancy worldwide and the third leading cause of cancer related death, exceeded only by cancers of the lung and stomach [1], [2]. HCC prevalence is higher in sub-Saharan Africa, central and Southeast Asia.

Surgery is the only potentially curative treatment for HCC. In carefully selected patients, resection and transplantation in fact, allow a 5 years survival ranging from 60 to 70%, and should be considered as a first treatment option in this setting [3].

Unfortunately most patients in Western countries present with an intermediate or advanced HCC at diagnosis with the consequent inability to use curative treatments. These patients are therefore candidates to palliative therapies such as arterial embolization, chemoembolization (TACE) and systemic treatment [4]. Only recently the molecular targeted drug, Sorafenib, has been introduced among the therapeutic options for these patients [5]–[8].

TACE represents a crucial treatment option for HCC, however comparing clinical findings resulted often hampered by the considerable variability in patients selection criteria and modalities of execution of therapy [9]–[12]. However, global results for TACE are still unsatisfactory, with only a small proportion of patients benefiting from these procedures. The molecular mechanism that accounts for treatment failure is not clear [13], [14]. It is possible that some adaptive responses to hypoxia may represent a key factor for resistance. Induction of tumor hypoxia combined with chemotherapy by transcatheter arterial chemoembolization has been widely used in treating unresectable HCC.

Hypoxia represents a clinical biological mechanism for treatment resistance in cancer cells via the formation of new blood vessels. Furthermore, a growing body of evidence indicates that hypoxia might actually promote cancer development.

There are significant differences between energy metabolism of cancer cells and that of normal tissues. Cancer cells maintain high aerobic glycolytic rates and produce high levels of lactate and pyruvate, a phenomenon known historically as the Warburg effect [15].

Lactic dehydrogenase (LDH), which is a glycolytic enzyme, composed of four polypeptide chains, each one encoded by separate gene (M and H), exists in various types of human tissue and neoplasms. LDH is a key enzyme in the conversion of pyruvate to lactate under anaerobic conditions [16]. Five isoforms of LDH have been identified as a result of the five different combinations of polypeptide subunits [17]. In preclinical models up-regulation of LDH has been suggested to ensure both an efficient anaerobic/glycolytic metabolism and a reduced dependence on oxygen under hypoxic conditions in tumor cells. Altered serum levels of LDH has also been reported in patients with HCC.

The biological link between hypoxia, LDH levels and the tumor-driven angiogenesis pathway through the abnormal activation of the hypoxia inducible factor 1 (HIF-1) is well established. The biological activity of HIF-1 is determined by the expression and activity of the HIF-1α subunit [18]. HIF-1α is an essential factor that upregulates a series of genes involved in glycolytic energy metabolism, angiogenesis, erythropoiesis and cell survival [19]. Hypoxia in the tumor microenvironment is sufficient to activate HIF-dependent expression of several downregulated genes [20]. These include genes encoding for vascular endothelial growth factor, erythropoietin and many enzymes involved in glucose, iron, and nucleotide metabolism [21].

Although links among these factors are well known, their translation into clinical practice is still poorly investigated. The aim of our analysis is to assess the prognostic role of LDH in a population of HCC patients, treated with TACE.

## Methods

### Patients selection

We retrospectively analyzed a population of HCC patients, treated with TACE (lipiodol or drug-eluting microspheres) from 2002 to 2010, at our institution. The study included all patients consecutively treated with TACE (in our institution, patients were treated with TACE with lipiodol from 2002 until 2006 and with TACE with microspheres from 2007 to 2010). Patients were classified according to ECOG PS (Eastern Cooperative Oncology Group performance status) and were staged using different staging systems: Child-Pugh, BCLC (Barcelona Clinic Liver Cancer), Okuda, MELD (Model for End-Stage Liver Disease), MELD-Na (Model for End-Stage Liver Disease – Sodium).

We recorded LDH serum levels pre- (within 1 month prior to treatment) and post-treatment (within one month after). LDH serum levels were determined according to IFCC (International Federation of Clinical Chemistry and Laboratory Medicine) method. The assay has been conducted in Our Institution Laboratory certified for Quality control according to the present rules in Europe (ISO 9001:2008). Patients were divided into two groups, according to LDH serum concentration registered before TACE. First group included patients with pretreatment LDH≤450 U/l, whereas the other group included patients with pretreatment LDH>450 U/l. LDH serum levels cut-off has been set to 450 U/l, because it is the upper limit of normality in blood specimens in our Institution. Patients were, also, classified according to any variation in LDH serum levels pre- and post-treatment (increased vs. decreased).

The A.O.U. “Ospedali Riuniti” of Ancona Ethical Committee approved the analysis. Patients gave written consent for their clinical information to be anonymously stored in the hospital database and used for research.

### Clinical outcome evaluation and statistical analysis

Treatment response was assessed through computed tomography (CT) and magnetic resonance imaging (MRI), alpha-fetoprotein (α-FP) assay, performed after one month of treatment and then every 3 months, according to the new RECIST criteria (New Response Evaluation Criteria in Solid Tumors 1.1). Radiological images were reviewed in double-blind by two radiologists.

The distribution curves of survival and time to progression were estimated using the Kaplan-Meier method. Overall survival (OS) was calculated as the time interval between the date of the procedure and the date of death or last follow-up. The time to progression (TTP) was calculated as the time interval between the date of the procedure and the date of progression or last follow-up.

The clinical variables analyzed were: gender (male vs. female), age (≤69 years vs. >69 years), ECOG PS (0–1 vs. 2–3), the Child-Pugh score (A vs. B), BCLC stage (A vs. B–C), Okuda stage (I vs. II vs. III), the MELD score (≤10 vs. 11–15 vs. >15), the MELD-Na score (≤10 vs. 11–15 vs. >15), the type of TACE (TACE or precision TACE; Lipiodol or drug eluting microspheres).

The association between variables was estimated using the chi-square test. Any differences between the groups were considered significant if the significance level was less than 0.05.

## Results

One hundred and fourteen patients were available for our analysis: 98 (86%) males and 16 (14%) females. Median age was 69 years (range 49–89) (Table 1). Eighty-four patients (74%) showed pretreatment LDH serum levels below the cut-off, while 30 (26%) were found above the chosen cut-off. Sixty-two patients (54%) showed decreased LDH serum levels after treatment, while in 52 (46%) this value increased.

**Table 1:** Clinical variables examined resulted balanced between the group of patients with high lactic dehydrogenase (LDH) serum levels (LDH>450 U/l) and those with a low value (LDH<450 U/l) and in those with a decreased or increased LDH serum level after transarterial-chemoembolization (TACE) treatment.

Clinical Variables		LDH<450 U/l	LDH>450 U/l	Total	LDH decreased	LDH increased	Total
Patients		84	30	114	62	52	114
Gender	Male	76	22	98	50	48	98
	Female	8	8	16	12	4	16
Median Age	<69	42	12	54	30	22	52
	>69	42	18	60	32	30	62
ECOG	0	28	10	38	21	17	38
	1	56	20	76	41	35	76
Child-Pugh	A	51	12	63	35	28	63
	B	33	18	51	27	24	51
BCLC	A–B	30	14	44	22	20	42
	C	54	16	70	40	32	72
Okuda	1	50	15	65	38	25	63
	2	34	15	49	24	27	51
MELD	<10	57	23	80	36	37	73
	10–15	27	7	34	26	15	41
MELD-Na	<10	39	17	56	38	25	63
	10–15	45	13	58	24	27	51

ECOG PS (Eastern Cooperative Oncology Group performance status), BCLC (Barcelona Clinic Liver Cancer), MELD (Model for End-Stage Liver Disease), MELD-Na (Model for End-Stage Liver Disease – Sodium).

No statistically significant differences were found between the groups of patients for all clinical characteristics analyzed (gender, median age, performance status ECOG, staging systems) (Table 1). Moreover, among the same groups, no significant differences were found in terms of objective responses (Table 2) and according with the type of TACE performed (lipiodol or drug-eluting microspheres).

**Table 2:** Objective response evaluated with RECIST criteria (New Response Evaluation Criteria in Solid Tumors 1.1), no statistically significant difference was found between the groups of patients analyzed: lactic dehydrogenase (LDH) major or minor of 450 U/l and LDH decreased or increased after treatment.

Objective Responce	LDH<450 U/l	LDH>450 U/l	Total	LDH decreased	LDH increased	Total
CR	19	5	24	13	9	22
PR	26	11	37	18	13	31
SD	19	3	22	21	14	35
PD	20	11	31	10	16	26

**Figure 1:**
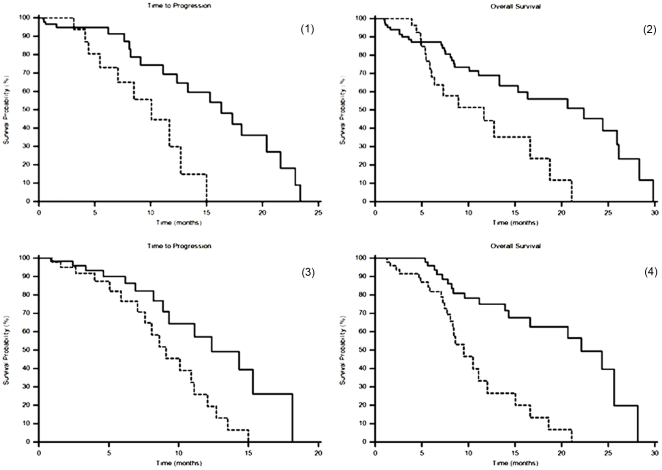
Survival analysis according to lactic dehydrogenase (LDH) serum levels in HCC patients undergoing transarterial-chemoembolization. Panel 1) Median time to progression (TTP) according to LDH serum levels: LDH≤450 U/l (–––), LDH>450 U/l (--------) (p = 0.0085). Panel 2) Median overall survival (OS) according to LDH serum levels: LDH≤450 U/l (–––), LDH>450 U/l (--------) (p = 0.0049). Panel 3) Median TTP according to the LDH serum levels variations pre- and post-treatment: LDH decreased post-treatment (–––), LDH increased post-treatment (--------) (p = 0.0087). Panel 4) Median OS according to the LDH serum levels variations pre- and post-treatment: LDH decreased post-treatment (–––), LDH increased post-treatment (--------) (p<0.0001).

In patients with LDH values below 450 U/l median time to progression (TTP) was 16.3 months, whereas it was of 10.1 months in patients above the cut-off. This difference was found to be statistically significant (p = 0.0085) (Figure 1, panel 1). Accordingly median overall survival (OS) was 22.4 months and 11.7 months in group A and B respectively (p = 0.0049) (Figure 1, panel 2). In patients with decreased LDH values after treatment median TTP was 12.4 months, and median OS was 22.1 months, whereas TTP was 9.1 months and OS was 9.5 in patients with increased LDH levels (TTP: p = 0.0087 Figure 1, panel 3; OS: p<0.0001 Figure 1, panel 4).

## Discussion

The clinical management of HCC is becoming increasingly challenging along with the growing availability of therapeutic options. Moreover the typical HCC patient has, in most cases, two different diseases, cancer and the underlying liver disease, both heavily influencing patients clinical outcome. In this setting patients stratification may represent a crucial factor for the choice of the appropriate treatment strategy for the appropriate patient. In fact, although many staging systems have been proposed, a relevant degree of heterogeneity can still be seen in the same sub-group of patients making treatment selection highly demanding.

In our experience, LDH serum levels seemed able to predict clinical outcome in terms of TTP and OS for HCC patients undergoing TACE. Globally our data indicated that LDH levels might identify patients with distinctive prognosis within the same staging group.

These findings are in accordance with previously published analyses suggesting a relationship between LDH levels and a worse outcome in different tumor types.

In colorectal cancer patients LDH up-regulation was in fact associated with an increased risk of nodal and distant metastases and high LDH serum levels have been shown to correlate with a decreased median overall survival [22], [23].

A strong association has also been demonstrated between the expression of LDH, in particular the LDH-5 isoform and an aggressive phenotype in gastric cancer [24].

This apparently enhanced tumor aggressiveness often determining worse prognosis in cancer patients showing high LDH levels has been traditionally correlated with molecular mechanism underlying tumor hypoxia and angiogenesis.

The possible link between LDH levels and tumor angiogenesis has been indicated in 2 different clinical trials (the CONFIRM 1 & 2 trials) investigating PTK/ZK (vatalanib), an oral inhibitor of VEGF (vascular endothelial growth factor) receptors, in first and second-line therapy of advanced colorectal cancer. Results from subsequent analyses from these trials in fact evidenced an improved median PFS with the use of PTK/ZK in patients with high serum LDH levels, thus suggesting that tumor angiogenesis represent a key crucial event in presence of high LDH levels [25], [26].

Increased levels of VEGF have been associated with tumor grade and with an inferior overall survival in HCC, apparently indicating that angiogenesis may represent a key-factor also for these patients [27], [28].

We also demonstrated that LDH serum levels variations (pre- and post-treatment) might correlate with clinical outcome in HCC patients. These findings seem to suggest that the biological phenomenon underlying LDH serum levels is a dynamic one and may be influenced by medical treatment. Recently Kohles et al. showed a possible prognostic role of pretreatment LDH serum levels in HCC patients undergoing TACE [29], confirming our hypothesis. We can then speculate that patients with high LDH pretreatment levels may be optimal candidates for clinical trial exploring a multimodality treatment approach including TACE and VEGF inhibitors.

After confirmation in larger analyses we believe that LDH should be considered as a relevant clinical variable to be included in the prognostic classification of HCC patients, with the aim to better define the most appropriate therapeutic strategy and to better stratify patients included in clinical trials.

Further studies testing the molecular and biological correlation between serum LDH levels and tumor angiogenesis are needed in both basic science and clinical arenas.
